# Integrating Adolescent Mental Health into HIV Prevention and Treatment Programs: Can Implementation Science Pave the Path Forward?

**DOI:** 10.1007/s10461-022-03876-2

**Published:** 2022-11-02

**Authors:** Judith Boshe, Veronica Brtek, Kristin Beima-Sofie, Paula Braitstein, Merrian Brooks, Julie Denison, Geri Donenberg, Elizabeth Kemigisha, Peter Memiah, Irene Njuguna, Ohemaa Poku, Sarah T. Roberts, Aisa M. Shayo, Dorothy E. Dow

**Affiliations:** 1 Department of Mental Health, Kilimanjaro Christian Medical Centre, KCMC Box 3010, Moshi, Tanzania; 2 Kilimanjaro Christian Medical College University, Moshi, Tanzania; 3 Duke Global Health Institute, 27701 Durham, NC USA; 4 Department of Global Health, University of Washington, Seattle, WA USA; 5 University of Toronto, Dalla Lana School of Public Health, Toronto, Canada; 6 College of Health Sciences, School of Public Health, Moi University, Eldoret, Kenya; 7 Department of Pediatrics, Craig Dalsimer Division of Adolescent Medicine, Children’s Hospital of Philadelphia, 19104 Philadelphia, PA USA; 8 Department of International Health, Johns Hopkins Bloomberg School of Public Health, Baltimore, MD USA; 9 Center for Dissemination and Implementation Science, Department of Medicine, University of Illinois at Chicago, Chicago, IL USA; 10 Faculty of Interdisciplinary Studies, Mbarara University of Science and Technology, Mbarara, Uganda; 11 Division of Epidemiology and Prevention, Institute of Human Virology, University of Maryland School of Medicine, Baltimore, MD USA; 12 Research and Programs, Kenyatta National Hospital, Nairobi, Kenya; 13 New York State Psychiatric Institute, HIV Center for Clinical and Behavioral Studies, Columbia University, 10032 New York, NY USA; 14 Women’s Global Health Imperative, RTI International, Berkeley, CA USA; 15 Department of Pediatrics, Infectious Diseases division, Duke University Medical Center, KCMC Box 3010, Box 102346, 27701 Durham, NC USA

**Keywords:** Adolescent, Mental health, HIV, Sub-Saharan Africa, Implementation science research

## Abstract

Adolescent mental health (AMH) is a critical driver of HIV outcomes, but is often overlooked in HIV research and programming. The implementation science Exploration, Preparation, Implementation, Sustainment (EPIS) framework informed development of a questionnaire that was sent to a global alliance of adolescent HIV researchers, providers, and implementors working in sub-Saharan Africa with the aim to (1) describe current AMH outcomes incorporated into HIV research within the alliance; (2) identify determinants (barriers/gaps) of integrating AMH into HIV research and care; and (3) describe current AMH screening and referral systems in adolescent HIV programs in sub-Saharan Africa. Respondents reported on fourteen named studies that included AMH outcomes in HIV research. Barriers to AMH integration in HIV research and care programs were explored with suggested implementation science strategies to achieve the goal of integrated and sustained mental health services within adolescent HIV programs.

## Introduction

There are more adolescents (10–19 years of age) living in the world today than at any other point in history [1]. Adolescent health and wellbeing are critical to our collective future; however, the COVID-19 pandemic has brought heightened attention to the striking adolescent mental health crisis, particularly among the most vulnerable adolescents at high risk for acquiring or living with HIV [2, 3]. Despite increasing research demonstrating that mental health is a critical mediator of HIV outcomes, the integration of mental health services remain largely absent from adolescent HIV prevention and care [4, 5]. Implementation science is the study of methods to promote the systematic uptake of research findings and other evidence-based practice into standard of care to improve the quality and effectiveness of health services [6]. Implementation science strategies can help pave the path to sustainable integration of mental health interventions poised to address increasing mental distress, to promote mental wellness, and to improve adolescent HIV prevention and treatment outcomes.

Prevalence studies show that mental health challenges affect approximately one-quarter to one-half of adolescents living with HIV (ALWH) [7]. Depression is a leading cause of illness and disability among adolescents, and suicide is the third leading cause of death worldwide for those 15–19 years of age with even higher rates of suicidal ideation and self-harm among adolescents living with HIV, often due to forms of HIV-related stigma [8, 9]. Major advances in HIV prevention and treatment technologies have been realized, yet attention to mental health and the negative consequences of mental health distress in HIV prevention, care engagement, and treatment adherence has lagged behind [10].

The developmental period of adolescence is marked by tremendous brain development, dramatic hormonal and physical changes that may partially explain adolescent’s tendency for risk taking, substance use, and the emergence of mental health distress [11]. In addition to the dramatic biologic changes, their social environments are rapidly changing. Peer networks and social connection become priorities and can influence decision making [12, 13]. ALWH have the added burden of navigating peer and romantic relationships while living with a stigmatizing and sexually transmittable infection that currently requires adherence to daily treatment. These factors contribute to worse HIV outcomes among adolescents compared to children or adults [14–16].

Adolescent mental health (AMH) is defined by the World Health Organization as a state of well-being resulting in a positive sense of identity, the ability to manage thoughts and emotions, build social relationships, and learn and acquire an education that enables full participation in society [17]. AMH broadly encompasses both well-being and mental illness and the continuum between them [18]. Approximately 50% of mental health disorders appear by the age of 14 and 75% by the age of 24 years [19, 20]. When mental distress (ranging from psychological distress to diagnosable mental illness) goes unrecognized or untreated it may lead to high-risk behaviors such as substance misuse, sex without a condom, or poor antiretroviral therapy adherence that increase the risk of HIV acquisition or transmission, and in extreme instances, self-harm or suicide [21, 22]. Recognizing mental distress early is critical to take advantage of the neuroplasticity of the adolescent brain and to strengthen positive coping strategies and neurologic pathways that maximize one’s ability to manage distress [23]. Reinforcing positive adaptive behavior patterns that shape social and emotional habits early in adolescence can help ALWH overcome adversity and maintain long-term mental health, self-efficacy, self-esteem, and resilience [8, 24].

Evidence-based interventions have clearly demonstrated efficacy to improve mental health with psychotherapy, medication, or a combination of the two [25, 26]. Research has also shown that addressing mental distress may lead to improved adherence and HIV outcomes for people living with HIV [27]. Yet, establishing efficacy of an innovation does not guarantee its uptake into routine use. Despite an increasing body of evidence describing the importance of addressing AMH in HIV prevention and treatment, integration of AMH into routine programming remains rare. [5, 10, 28–30]. This is known as the “know-do gap” in implementation science research. The implementation science framework, Exploration, Preparation, Implementation, Sustainment (EPIS), offers guidance to facilitate understanding of the diverse determinants (barriers and facilitators) at multiple levels (e.g., individual, health care setting, national policy) that impact adoption, acceptability, appropriateness, and sustainability of evidence-based interventions [31]. Implementation strategies must be used to overcome identified barriers and enhance facilitators to increase the uptake of evidence-based clinical innovations [32].

This research article provides an initial evaluation of the “research to public health impact” approach [33] towards integrating AMH in HIV research and care programs through the lens of a global alliance of adolescent HIV prevention and treatment researchers, providers, and implementors who work in sub-Saharan Africa (SSA). Informed by the EPIS framework, we sought to (1) describe how AMH is incorporated in their current HIV research; (2) evaluate contextual determinants influencing the integration of AMH into adolescent HIV research and care programs; and (3) describe current AMH screening and referral practice within HIV care programs in the sub-Saharan African contexts in which alliance members work.

## Methods

### Sample

This study leveraged two global scientific networks (28 unique teams) designed to improve the prevention and care of adolescent HIV in resource constrained settings: the Prevention and Treatment through a Comprehensive Care Continuum for HIV-affected Adolescents in Resource Constrained Settings (PATC^3^H) and the Adolescent HIV Implementation Science Alliance (AHISA) funded by the NIH and led by the Fogarty International Center [34]. AHISA includes 26 independent research teams focused on adolescent HIV prevention and treatment in SSA with relevant NIH funded research projects [35]. PATC^3^H includes eight research teams focused on combination interventions to improve health outcomes among adolescents with or at risk for HIV, seven of which are located in SSA [36].

### Study Design

The study leveraged the robust AHISA and PATC^3^H networks, hence forth referred to as “the alliance”, to provide a unique perspective from researchers, implementors, and clinicians in SSA to understand the current research and implementation strategies being used to integrate AMH in HIV research and care. This was accomplished using the EPIS framework to guide questionnaire development and analysis. EPIS is a comprehensive implementation science framework that proposes four consecutive phases to facilitate the process of bringing evidence to practice [37]. We initially focused on the “exploration phase” by comprehensively surveying “the alliance” about AMH research that is being conducted in each team, including the evidence-based approaches being deployed, project locations, and common measurement tools. Next, we asked research teams to describe the inner and outer context determinants (barriers, gaps, constraints) impacting the delivery and integration of AMH in HIV prevention and treatment at their sites. [38]. Responses were considered part of the “preparation phase” as teams indicated how they planned to mitigate and leverage the context-related barriers. Building on study responses, we identified implementation strategies consistent with Powell’s Expert Recommendations for Implementing Change (ERIC) compilation of 73 discrete strategies [39] towards realizing the final stages of “implementation” and “sustainment” of the EPIS framework.

Responses were interpreted based on EPIS constructs [37]. The “outer context” in this paper refers primarily to the sociopolitical context at the country level including policies and funding streams that impact priorities, but also the needs and characteristics of adolescents who seek HIV prevention or treatment services. Outer context decisions often influence the “inner context” defined in this paper as the organization, hospital, or clinical setting and staff attitudes and beliefs. Innovation factors incorporate characteristics of the intervention and flexibility or adaptability for innovation to maximize implementation. In this case, we are considering barriers to integrating evidence-based AMH interventions into existing care programs connected to “the alliance” in SSA. Bridging factors are defined as spanning or bridging the “outer context” namely the community (characteristics and needs of adolescents) and the “inner context” including the institution, hospital, or clinic, but were largely beyond the scope of the questionnaire.

### Questionnaire

The questionnaire took approximately 5–15 min to complete. The initial questions queried whether “the alliance” incorporated AMH into their adolescent HIV research over the last five years. Members who reported measuring AMH were asked to provide the study title(s), research setting(s) (clinic/hospital, community, school, faith, other), country(ies) location(s), mental health constructs, and measurement tools. The authors sought additional study details via NIH REPORTER, Clinicaltrials.gov, Pubmed, and direct email with the principal investigators as needed. Next, all alliance teams were invited to complete a survey of open-ended questions to explore barriers, gaps, and constraints in AMH research and service provision in the context of HIV prevention and treatment programs regardless of whether they incorporated AMH in their adolescent HIV research.

The questionnaire was proposed at the annual joint AHISA/PATC^3^H meeting on February 12, 2021. Alliance teams agreed to participate and the questionnaire was designed, reviewed by all co-authors and pilot tested. Once revisions were incorporated, the questionnaire link was distributed to teams by email using the NIH list serve for the AHISA/PATC^3^H teams. The first email was sent on May 13, 2021 with a reminder sent on May 28, 2021.

### Data Management and Analysis

Data were captured and managed using REDCap electronic data tools hosted at Duke University [40, 41]. Survey results were analyzed using Stata version 17.0 (StataCorp, College Station, TX) and are presented as descriptive statistics with a qualitative component.

## Results

Eighteen of the 28 (64%) AHISA and PATC^3^H teams completed the survey, reporting on research conducted in 11 SSA countries (Botswana, Ghana, Kenya, Malawi, Mozambique, Nigeria, South Africa, Tanzania, Uganda, Zambia, Zimbabwe) which are home to over half of all adolescents living with HV globally [42] (Table 1). Half of respondents (n = 9) reported their primary adolescent HIV research focus was on HIV care and treatment. The other half focused on prevention, including pre-exposure prophylaxis (PrEP) and sexual and reproductive health (SRH). One respondent specifically mentioned utilizing implementation science to improve the continuum of HIV service delivery among adolescents.

**Table 1:** Reported Adolescent HIV Prevention and Treatment Research Incorporating Mental Health Outcomes

Study name	Country	Affiliation	Setting	Aim	Study Design	Mental health Objective	Constructs measured	Tool
Adolescent Friendship Bench adaptation and pilot in Botswana	Botswana [45]	AHISA	Community based and clinic/hospital	Aims are to assess the feasibility of a Problem Solving Therapy based lay counseling program using young adult lay counselors to improve mental health.	Pre-post design	Primary	Depression, emotional regulation, trauma, anxiety, suicide/self-harm, substance misuse, wellness	PHQ-9, SSQ Shona symptoms questionnaire Trauma-ex, GAD-7, AUDIT
Understanding and measuring the impact of stigma on PrEP adherence among adolescent girls and young women in Kenya: identifying targets for future interventions	Kenya [46]	Both	Community based and clinic/hospital	Improve understanding of stigma-related barriers to PrEP adherence	Mixed Methods	Exploratory	Depression, anxiety, wellness	PHQ-4, *RS-14
SMART: **S**trengthening **M**ental health **A**nd **R**esearch **T**raining ClinicalTrials.gov Identifier: NCT03081195	Kenya, Uganda and Ghana [76]	AHISA	School	A scale-up longitudinal experimental study that uses a mixed-methods, hybrid type II, effectiveness implementation design to test the effectiveness of an EBP, called Multiple Family Group (MFG) for school children, young adolescents 8–13 y/o	Randomized controlled trial, parallel	Primary	Depression, anxiety	SDQ, CDI (child depression inventory, Tennessee self-concept **Parent** CES-D, Brief Symptom Checklist, and parent stress index short form
​DiSC (Data-informed Stepped Care) ClinicalTrials.gov Identifier: NCT05007717	Kenya [77]	Both	Clinic/hospital	The data-informed stepped care (DiSC) intervention pairs stepped care with a clinical prediction tool to optimize limited resources and improve HIV care and treatment outcomes.	Randomized controlled trial, parallel	Exploratory	Depression, anxiety, substance misuse	PHQ-9, GAD-7, AUDIT
​​CombinADO (Combination intervention strategy to improve health outcomes for adolescents) ClinicalTrials.gov Identifier: NCT04930367	Mozambique [36, 78]	PATC3H	Clinic/hospital	Evaluate the effectiveness and monitor implementation of a community-informed multi-component intervention (“CombinADO strategy”), mental health screening and linkage to adolescent-focused mental health support	Cluster randomized controlled trial	Secondary	Depression, trauma, anxiety, suicide/self-harm, substance misuse, *stigma	PHQ-9, GAD-7, AUDIT,
Adolescent Coordinated Transition (ACT) to improve health outcomes among young people living with HIV in Nigeria ClinicalTrials.gov Identifier: NCT03152006	Nigeria [79]	AHISA	Community based Clinic/hospital	Evaluate the comparative effectiveness of ACT, a coordinated protocol for transitioning adolescents living with HIV (ALHIV) from pediatric to adult care (Intervention Group; IG) versus the usual abrupt transfer to adult care (Control group; CG) on rates of retention in care and viral suppression, and differences in perceived psychosocial wellbeing.	Cluster, randomized control trial	Secondary	Depression, wellness	*SRBBS, *MHC-SF, health locus of control
​ITEST (Innovative Tools to Expand Youth-Friendly HIV Self-Testing): Known in Nigeria as the 4 Youth by Youth project ClinicalTrials.gov Identifier: NCT04710784	Nigeria [36]	Both	Community based and school	Develop and evaluate novel youth-friendly HIV Self Testing services in Nigeria using open challenges and apprenticeship training informed by a participatory learning collaborative model to reach young Nigerians that remain undiagnosed for HIV and to facilitate linkage and retention in preventive services (includes STI testing/treatment, PrEP referral, condom use).	Stepped-wedge cluster randomized controlled trial	Exploratory	Depression, substance misuse, wellness	PHQ-9
​IMARA (Informed Motivated Aware and Responsible Adolescents and Adults) ClinicalTrials.gov Identifier: NCT04758390	South Africa [36, 80]	Both	Clinic/hospital	A pilot test of an empirically supported culturally adapted family-based HIV-prevention program,	Randomized controlled trial	Secondary	Depression, emotion regulation, trauma, anxiety	PHQ-9, PC-PTSD-5, DSM-5 Trauma RI, GAD-7, AUDIT
IMPAACT 2016 ClinicalTrials.gov Identifier: NCT04024488	South Africa, Botswana, Malawi, Zimbabwe [81, 82]	Both	Clinic/hospital	Evaluate whether an Indigenous Leader Outreach Model (ILOM) of trauma-informed cognitive behavioral therapy (TI-CBT) delivered by Indigenous Youth Leaders (IYL) is associated with improved mental health outcomes and ART adherence among youth living with HIV in resource-limited settings	Randomized controlled trial	Primary	Depression, emotional regulation; trauma; anxiety;	PHQ9, Trauma RI, GAD-7
MTN-034/REACH ClinicalTrials.gov Identifier: NCT03593655	SA, Uganda, Zimbabwe [83]	Both	Clinic/hospital	This study will evaluate the safety of and adherence to a vaginal matrix ring (VR) containing dapivirine and oral emtricitabine/tenofovir disoproxil fumarate (FTC/TDF) in adolescent and young adult females	Randomized controlled trial, crossover	Exploratory	Depression; substance misuse	CES-D, AUDIT-C
Establishing mental health needs for adolescents in Tanzania	Tanzania [47, 84, 85]	AHISA	Clinic/hospital	Understanding prevalence and associations between MHD and adherence, stigma, and disclosure	Cross-sectional study	Primary	Depression, emotional regulation, trauma, suicide/self-harm, substance misuse, *stigma	PHQ-9, SDQ, UCLA Trauma RI, Berger Stigma Scale (10 questions)
Sauti ya Vijana (SYV: The Voice of Youth) ClinicalTrials.gov Identifier: NCT02888288 (pilot) NCT05374109 (effectiveness trial)	Tanzania [43, 86]	AHISA	Clinic/hospital	Pilot evaluated feasibility and acceptability of a MH intervention to improve adherence and virologic suppression. Now an effectiveness trial	Pilot, Randomized controlled trial, crossover	secondary	Depression; Emotional regulation; trauma; substance misuse	PHQ9, SDQ, UCLA Trauma RI
Project YES! Youth Engaging for Success ClinicalTrials.gov Identifier: NCT04115813	Zambia [87–92]	AHISA	Clinic/hospital	Test the impact of a theory-based intervention that utilizes trained and paid HIV-positive youth peer mentors (YPMs) to deliver the intervention consisting of monthly one-on-one and small group sessions with AYA, ages 15 to 24 years. Optional caregiver support groups are incorporated.	Randomized controlled trial, crossover	Other, please explain	Depression, suicide/self-harm *Internalized stigma	Hopkins depression scale, AUDIT, Kalichman stigma scale, ART self-efficacy
Family Connections ClinicalTrials.gov Identifier: NCT05358795	Zambia [93]	AHISA	Clinic/hospital	Family-focused group intervention for adolescents and young adults living with HIV (AYALWH) and their caregivers with the aim to increase social and family support and decrease self-stigma so AYALWH may improve their medication adherence and achieve an undetectable viral load	Cluster, randomized controlled trial	Secondary	Depression, emotional regulation; trauma; substance misuse; Suicide/self-harm; wellness; other	PHQ-9, PROMIS Pediatric Cognitive Function; Difficulties in Emotion Regulation Scale (DERS-SF); Child and Youth Resilience Measure (CYRM; Adult Resilience Measure; modified Kalichman internalized stigma scale

AHISA: Adolescent HIV Prevention and Treatment Implementation Science Alliance; PATC^3^H: Prevention and Treatment through a Comprehensive Care Continuum for HIV-affected Adolescents in Resource Constrained Settings. Both: investigators are members of both the AHISA and PATC^3^H network. Investigators from these networks work in 11 distinct and high-HIV burden sub-Saharan Africa countries: Botswana, Ghana, Kenya, Malawi, Mozambique, Nigeria, South Africa, Tanzania, Uganda, Zambia, Zimbabwe. Questionnaires not mentioned in the text: Mental Health Continuum-Short Form (MHC-SF); Sexual risk behavior beliefs and self-efficacy (SRBBS) survey (developed by Basen-Engquist et al.)

Resilience Scale-14 (RS-14); Center for Epidemiologic Studies Short Depression Scale (CES-D)

### Exploration Phase: Is AMH Incorporated into Alliance Members’ HIV Research?

Twelve of 18 respondents (67%) reported measuring AMH in their research projects within the last five years; four of whom reported two different projects (1 duplicate removed and one not listed as no title was provided) for a total of 14 uniquely named studies (Table 1). Eleven of 14 studies (79%) were randomized controlled trials. Three studies were evaluating a mental health intervention to improve mental health either as a primary outcome or secondary outcome as a mediator of ART adherence and HIV outcomes [43–45]. Other studies reported using mixed methods studies with mental health as an exploratory aim [46] or cross-sectional prevalence study with mental health as a primary aim [47]. In all, 64% (9/14) of projects included mental health as a priority construct (primary or secondary objective) in their research study.

Depression, measured in all studies, was the most common mental health construct evaluated. The nine-question Patient Health Questionnaire (PHQ-9) was used in 10 of 14 studies with alternative measures including PHQ-4, Child Depression Index (CDI), Center for Epidemiologic Studies-Depression (CES-D), and Hopkins Depression Scale (Table 1). Anxiety was measured in half of the studies (7/14), five of which used the Generalized Anxiety Disorder Screening tool (GAD-7). The Alcohol Use Disorders Identification Test (AUDIT) was used to measure substance misuse in six studies (42%). A third of respondents (33%) reported including measures of mental wellness, but there was less consistency in measurement tools. Examples included the Mental Health Continuum Short Form [48], the Resilience Scale-14 [49] and the Child and Youth Resilience Scale [50]. Internalized stigma was listed as an “other” mental health construct in three studies, underscoring the important association of HIV stigma with mental health.

*Preparation phase: What are the determinants (barriers, gaps, constraints) of AMH in HIV research and service delivery?*

**Fig. 1:**
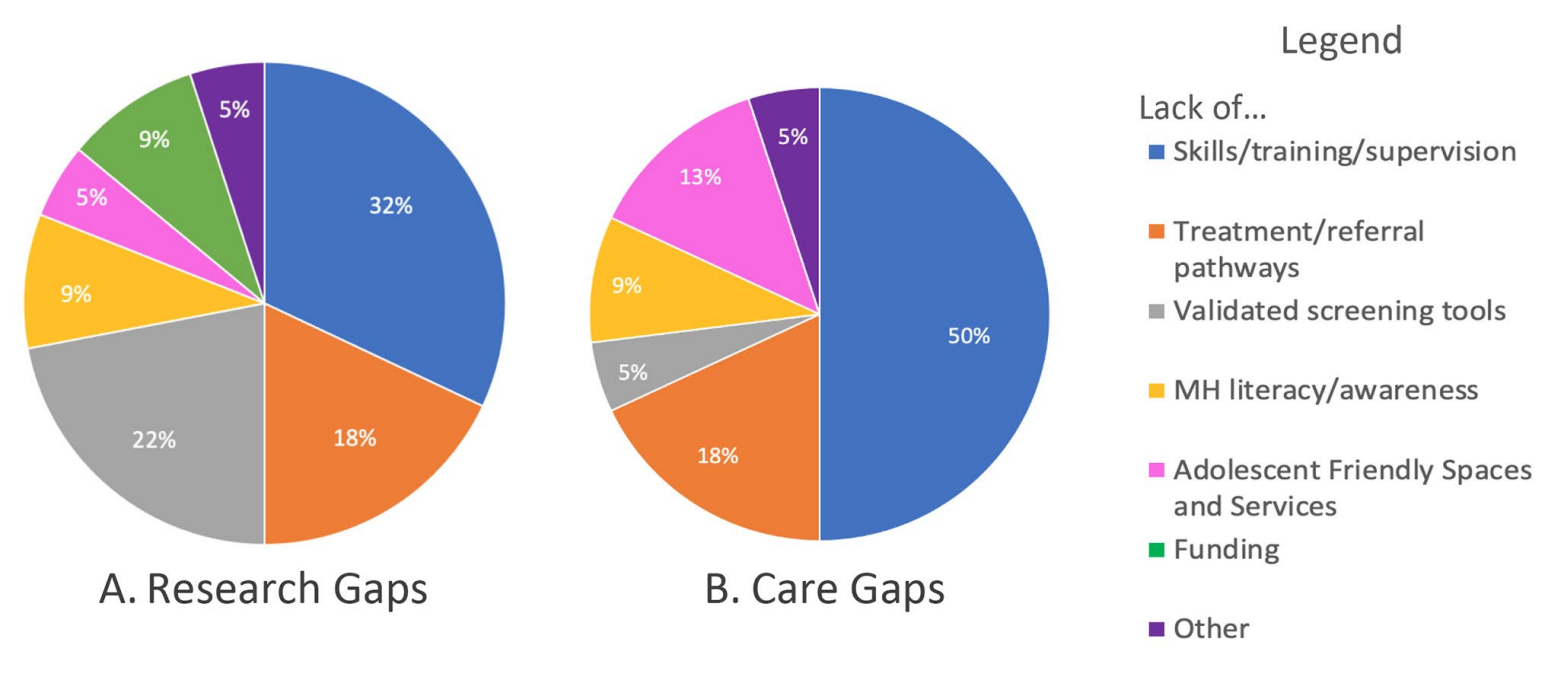
Questionnaire Responses to Barriers Incorporating Adolescent Mental Health into HIV Research and Care. Other (N=1) Research Gap: Institutional Review Board; Other (N=1) Care Gap: Integrating AMH into care without a specific named barrier

Fifteen of 18 respondents (83%) completed open ended questions about barriers to incorporating evidence-based AMH screening and interventions into HIV research and service delivery. All 15 respondents reported in-country barriers, gaps, and constraints to integrating AMH in the context of HIV prevention and treatment. The reported barriers in HIV research versus HIV care are compared in Fig. 1 and were harmonized across domains. The greatest barrier to both research and care was lack of personnel with skills or training in mental health. The second greatest barrier in both research and care was lack of treatment and referral pathways. Limited mental health literacy was named as a barrier by 9% of respondents for both research and care. Lack of validated screening tools and funding were more frequently named a barrier related to research. Lack of adolescent friendly spaces and services was more frequently reported as a barrier related to care.

**Fig. 2:**
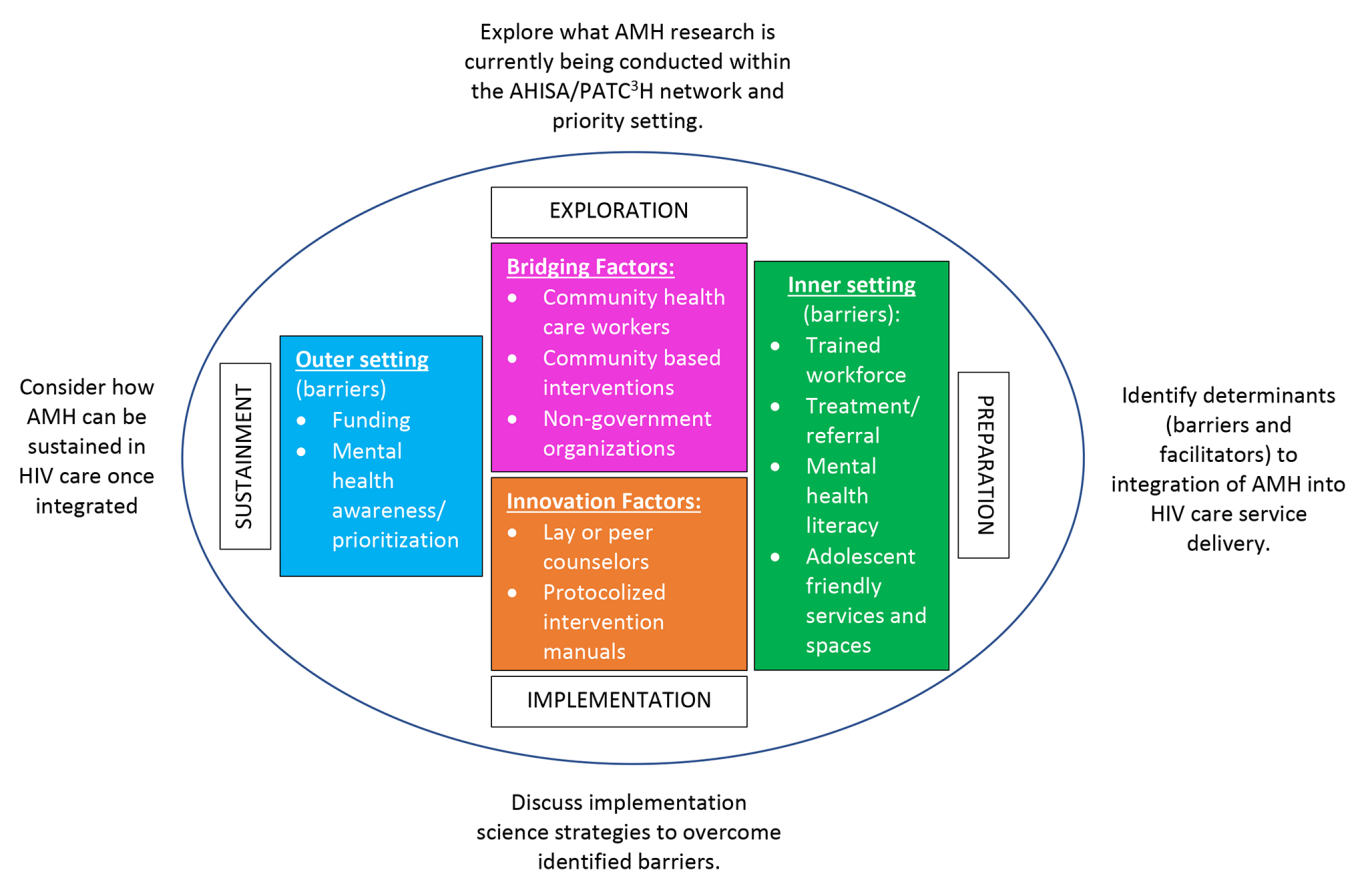
Study aims (Exploration and Preparation), findings (inner colored boxes), and discussion points (implementation and sustainment) mapped onto the EPIS framework

Barriers were mapped on the EPIS framework (Fig. 2). The most common barriers concerned the “inner setting”. Specifically, lack of an appropriately trained mental health workforce. As one respondent wrote “*Staff training; capacity for screening, diagnosis, treatment; lack of good treatment options available in many settings*” were the main barriers to integrating AMH into HIV care.

A proposed implementation strategy was leveraging evidence-based research on the success in training paraprofessionals such as lay counselors and peers to provide AMH services to address insufficient mental health care provider capacity. It was noted, however, that though this strategy could facilitate care for many, formally trained professionals remain necessary to treat adolescents with more severe mental illness and capacity must be built to address this need.

As one respondent wrote: “*Simple ‘layperson counselling’ is not enough to deal with it once there is pathology.”* Another respondent suggested that acquiring and maintaining competencies in AMH could be an important metric to ensure proficiency among those providing care.

Respondents also noted the lack of access to adolescent friendly services and spaces, whereby adolescents access an environment where they feel welcome, receive care at a convenient time, are free of judgment, and have confidence in staff. There was also mention of the lack of a consistent mental health definition and limited mental health literacy and awareness. Respondent’s noted that “*Many researchers, healthcare workers, and adolescents themselves know very little*,” and “*There is not enough awareness of the problem.”*

Lack of funding was the main outer setting barrier mentioned by respondents, including “*funding gaps*” and “*lack of investment*.” Lack of awareness of AMH issues and the impact of mental health on HIV outcomes was also noted to be lacking at the leadership level. For example, one respondent commented that*“There is no provision of mental health services within the context of HIV in many, many countries - especially outside of the capitol. I think trauma, suicide, alcohol and substance use all need program strengthening in the context of HIV care.”*

Respondents were asked about current practice and if adolescents who seek HIV prevention (such as care for sexually transmitted infections or PrEP) or treatment services (such as antiretroviral therapy refills) are screened for mental health in the SSA country in which they work. Screening using the PHQ-9 was reported in Botswana and Uganda, but respondents from other countries were either unsure or reported that screening was not routine. Only eight of 15 respondents reported a referral pathway for adolescents with mental distress in routine care. The majority of referrals were to a social worker, some to a psychologist, and few reported the existence of psychiatry, although these were only in larger urban, academic settings. When a referral process was present, respondent’s noted that they were often incomplete but provided a starting place: “*it is not very much or very effective - but there is something to build off of.”* Of the eight respondents who reported an existing referral system, five described additional barriers to access including the stigma associated with seeking mental health care and the cost of the appointment or transportation to the mental health clinic and noted how alternative strategies might be needed. For example, one participant commented that the*“use of para-professionals and peers may work better; Youth often don’t show up due to stigma/cost and the number of appointments available are extremely limited.”*

## Discussion

AMH is increasingly realized as a contributor to HIV outcomes and overall well-being for adolescents at risk or living with HIV, but significant gaps integrating AMH into HIV prevention and treatment research and care persist. This exploratory investigation examined the application of AMH research in the context of HIV prevention and treatment in SSA using an EPIS-informed questionnaire. Characterization of barriers, gaps, and constraints generated important directions for implementation strategies that fall within the ERIC taxonomy and can be leveraged to strengthen the integration of AMH into HIV prevention and care.

Alliance members reported on 14 studies, primarily RCTs, across 11 SSA countries. These studies helped summarize and “explore” evidence-based measurement tools, strategies, and interventions that may be a good fit for addressing local AMH needs in HIV prevention and care delivery in SSA. Importantly, a key indication that AMH was a scientific priority was that all studies measured symptoms of depression, most commonly using the PHQ-9, and this was also the measure reported for screening in routine care in Botswana and Uganda. The PHQ-9, and the even shorter PHQ-2 have been endorsed as a screening tool of choice [51, 52], but validation among adolescent populations in SSA is often extrapolated from other contexts and adult populations [53]. Prevalence studies suggest that between 10 and 50% of adolescents and young adults living with HIV in SSA have symptoms of moderate to severe depression and that such symptoms predict poorer prevention and HIV treatment outcomes [7, 47, 53, 54]. Importantly, other forms of distress including anxiety, trauma, neglect and abuse were also measured in many studies within “the alliance” (Table 1). Research has shown that adverse childhood experiences, along with inherited, social, and environmental factors also drive mental health distress and predict poorer health outcomes [55–57]. Mental wellness factors were evaluated in only a third of reported studies in the questionnaire and are an important direction for future research. The importance of coping skills, resilience, and quality of life as components of mental wellness on the mental health continuum should not be overlooked [18].

Several AMH interventions for youth living with HIV, all of which utilize peer youth leaders, are being evaluated within the alliance [43–45, 58, 59]. If proven effective, scale, sustainability, and integration of evidence-based AMH interventions will require adaptation to new contexts and engagement of local stakeholders for sustainment. Protocolizing intervention manuals may help ensure fidelity, but implementation strategies that “promote adaptability” by identifying the ways an AMH intervention can be tailored to meet the local needs while naming the critical elements necessary to preserve fidelity will be necessary. Use of adaptation tools, such as FRAME [60] will help track adaptations and preserve critical elements when interventions are scaled to new contexts.

One implementation strategy for optimizing adaptation includes “assessing for readiness and identifying barriers and facilitators” through early engagement by the research teams with the inner context (characteristics of the implementing organization) and outer context (national policy and financing landscape) to help facilitate early adoption and improve the chances of sustainable integration. The “Theory of Change” model used by the Friendship Bench, a mental health intervention from Zimbabwe, described a successful process for early engagement of stakeholders towards successful, sustainable integration [61]. Use of mobile technology to deliver mental health interventions also shows promise in pilot studies, but may be limited by adolescent’s access to smart phones and internet bundles [62]. Digital technologies and mobile platforms, though not perfect, provide solutions to challenges of scale, cost, and mitigate against the challenges of in-person contact faced during the COVID-19 pandemic (see Zanoni mHealth systematic review in this supplement for additional detail).

The most commonly cited barrier, reflected within the “inner setting”, was a paucity of trained providers. Lack of treatment and referral options also pose a dilemma regarding the ethics of AMH screening when little or no treatment is available. The UNAIDS Report on Integrating Mental Health and HIV interventions states, “Screening should take place only when clinical services are available to provide clinical assessment to verify a diagnosis and to provide any needed care” [63]. However, research describing the local prevalence of AMH in the context of HIV programs may help build awareness and demonstrate a need for programs that prioritize capacity building of a trained and integrated mental health care workforce. Bridging factors, such as a non-government organization teaching brief interventions to medical professionals or community health workers, can help provide hopefulness, support, and self-efficacy to help bridge the gap to professional mental health care [64, 65]. The Common Elements Treatment Approach (CETA) offers free, online, evidence-based resources that have been rigorously evaluated in SSA to train non-professionals to provide quality and effective mental health care [66]. The World Health Organization mhGap Community Toolkit is an another evidence-based model that leverages community resources to provide AMH care [67]. Thus, lack of professional services should not be a barrier to screening, rather screening should be viewed as a potential facilitator towards increasing mental health awareness and building mental health capacity to address the identified needs. Consistent with ERIC, these barriers point to several potential implementation strategies, such as: (i) “conducting educational meetings/outreach visits” to inform local providers of how to address AMH, (ii) “conducting ongoing training” in the evidence-based intervention, and (iii) “tailoring strategies” such as training paraprofessionals such as lay counselors, community health care workers, and peers.

Evidence-based interventions that specifically aim to build adolescent mental health capacity within HIV prevention and treatment programs using a cadre of trained peers is growing. In addition to interventions within the alliance (Table 1), “Let’s Talk” is a group-based intervention that holds promise to promote mental wellness and address mental health distress for HIV prevention among at risk adolescents and their caregivers in South Africa [68]. Zvandiri community adolescent treatment supporters (CATS) [69] are being trained in the successful Friendship Bench model [70], a non-protocolized, individual problem solving skills intervention to help adolescents living with HIV with mental health challenges. The TRUST framework was developed after evaluating the program from the perspective of the CATS and highlights the critical importance of close supervision and mental health support for peer counselors themselves as well as the need for additional referral pathways to accommodate more complex cases [71].

Another inner context barrier cited by respondents was lack of Adolescent Friendly Services and Spaces (AFSS) that are sensitive to adolescent psychosocial, mental health, and developmental needs and offer privacy. A study from Tanzania highlighted the government’s prioritization of AFSS, but found critical challenges including lack of a standard definition of AFSS, inconsistent quality of services provided, and inadequate reach to adolescents [72]. Similarly, a cross-sectional analysis of AFSS in South Africa demonstrated the need for implementation science approaches for sustaining quality standards [73]. Having a “one-stop shop” with non-judgmental providers trained in adolescent health with mental health care capacity was highlighted as an important next step in providing effective integrated care for AMH within HIV prevention and treatment programs.

In the outer context, funding gaps and failure to prioritize AMH were noted barriers. According to the WHO Mental Health Atlas, median government expenditure on mental health services in Africa in 2020 had risen to a mere $0.46 per capita, though data was only available for eight countries [74]. It has been reported that for every US$ 1 invested in treatment for depression and anxiety, there is a US$ 4 return of investment through better health outcomes [63, 75]. Funding for mental health and psychosocial support integrated into adolescent HIV prevention and treatment research and service delivery is critical to ending the AIDS epidemic, but without awareness and prioritization in the outer context, a dearth of funding and lack of resources is likely to persist. Linking outcome improvement to funding AMH services could be adopted and optimized within routine HIV care towards increased and sustained funding. ERIC implementation strategies such as “access new funding” to facilitate the implementation and “develop and organize quality monitoring systems” to monitor clinical processes and/or outcomes for the purpose of quality assurance and improvement can be utilized to tackle funding constraints.

Limitations of this study include inquiry of a specific alliance that may not generalize to all stakeholders across SSA. Within the AHISA/PATC^3^H networks, those passionate about AMH may have been more likely to respond to the questionnaire, thus findings may represent a biased sampling within the alliance. The determinants revealed during the study’s *Preparation* phase used open-ended questions and respondents did not cite facilitators, likely due the wording of the questionnaire. The *Implementation and Sustainment* components of EPIS were beyond the scope of the questionnaire, but were considered with regard to next steps in the discussion. Despite these limitations, the data provided by the questionnaire and the alliance portray important insight into the urgent need to incorporate AMH into HIV prevention and treatment research, and implementation strategies that are being used to innovate and bridge gaps towards integrating AMH into HIV prevention and care in the African setting.

## Conclusion

Implementation science can bring together key stakeholders—researchers, providers, policy makers, adolescents and families—to work together to critically explore evidence-based interventions, consider innovative strategies to address potential barriers and evaluate practical models that can be integrated and sustained into existing health care systems to improve AMH and adolescent HIV prevention and treatment outcomes.

## Data Availability

Provided at request to the corresponding author(s).

## Code Availability (software application or custom code)

Provided at request to the corresponding author(s).
